# Isolated Pulmonary Infective Endocarditis with Septic Pulmonary Embolism Complicating a Right Ventricular Outflow Tract Obstruction: Scarce and Devious Presentation

**DOI:** 10.1155/2013/746589

**Published:** 2013-09-09

**Authors:** Abdelrahmen Abdelbar, Raed Azzam, Kok Hooi Yap, Ahmed Abousteit

**Affiliations:** Department of Cardiothoracic Surgery, Manchester Royal Infirmary, Oxford Road, Manchester M13 9WL, UK

## Abstract

We present a case of a fifty-three-year-old male who presented with severe sepsis. He had been treated as a pneumonia patient for five months before the admission. Investigations revealed isolated pulmonary valve endocarditis and septic pulmonary embolism in addition to undiagnosed right ventricular outflow tract (RVOT) obstruction. The patient underwent surgery for the relief of RVOT obstruction by substantial muscle resection of the RVOT, pulmonary artery embolectomy, pulmonary valve replacement, and reconstruction of RVOT and main pulmonary artery with two separate bovine pericardial patches. He was discharged from our hospital after 6 weeks of intravenous antibiotics. He recovered well on follow-up 16 weeks after discharge. A high-suspicion index is needed to diagnose right-side heart endocarditis. Blood cultures and transesophageal echocardiogram are the key diagnostic tools.

## 1. Introduction 

Infective endocarditis involving the right side of the heart is an uncommon condition, which often involves the tricuspid valve [1]. It is even rarer to see isolated pulmonary valve endocarditis. This has led to the absence of definite guidelines to aid the management. In this particular case, pulmonary valve endocarditis was predisposed by undiagnosed right ventricular outflow tract (RVOT) obstruction. This had led to delayed diagnosis and, therefore, treatment.

## 2. Case Presentation 

A fifty-three-year-old man was referred to the cardiothoracic surgical team for consideration of surgery. Five months earlier he was diagnosed and treated by his general practitioner for what was thought to be pneumonia. He did not completely recover after a course of antibiotics. The patient's symptoms deteriorated and were resistant to antibiotics. He was therefore admitted to a local hospital. Early during the hospital stay, ongoing sepsis led to vasculitic rash in both lower limbs. He was transferred to a tertiary hospital for further workup and treatment plan. Past history included a spontaneously healed ventricular septal defect (VSD) during childhood but no further follow-up was done. He remained septic despite being on intravenous (IV) antibiotics. Successive blood cultures were negative which were thought to be the results of response to antimicrobial treatment. Antibiotics were changed according to the microbiology team advice, but the patient deteriorated after a brief response. Transesophageal echocardiogram (TOE) confirmed the diagnosis of pulmonary valve endocarditis with RVOT obstruction. The decision was made to operate on the patient to remove the source of sepsis. A high suspicion index is needed to diagnose right-side heart endocarditis. Blood cultures and TOE are the key diagnostic tools.

## 3. Diagnosis, Treatment, and Outcome 

**Figure 1:**
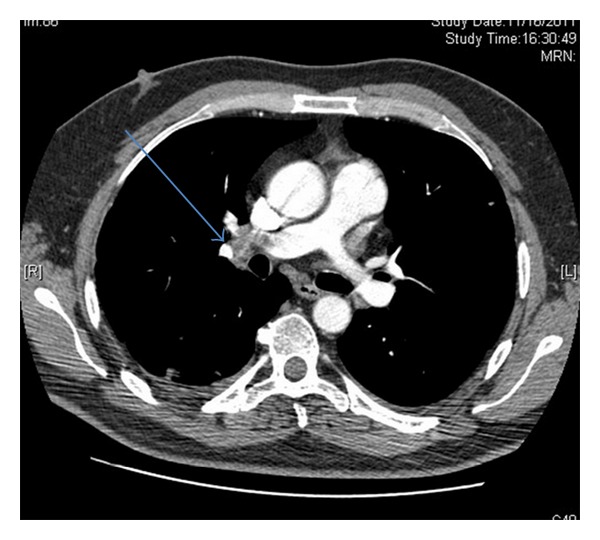
Preoperative axial CTPA showing pulmonary embolism (arrow) in the distal part of the right pulmonary artery.

Biochemical and haematological investigations showed a raised acute phase inflammatory marker with autoanticoagulation and deranged liver function. Only the first blood culture showed staphylococcus species. Subsequent microbiology cultures showed no growth, this was believed to be due to antibiotics. Computed tomography pulmonary angiography (CTPA) revealed right pulmonary artery embolism (Figure 1). Transthoracic echocardiogram (TTE) and TOE revealed RVOT obstruction with destroyed pulmonary valve. Besides, they had also excluded the presence of VSD.

As the sepsis was resistant to antibiotics, the decision was to operate on the patient to remove the septic emboli and correct the predisposing cause of sepsis. The patient underwent surgery for the relief of ROVT obstruction by substantial muscle resection of the RVOT, pulmonary artery embolectomy, pulmonary valve replacement, and reconstruction of RVOT and main pulmonary artery with two separate bovine pericardial patches.

**Figure 2:**
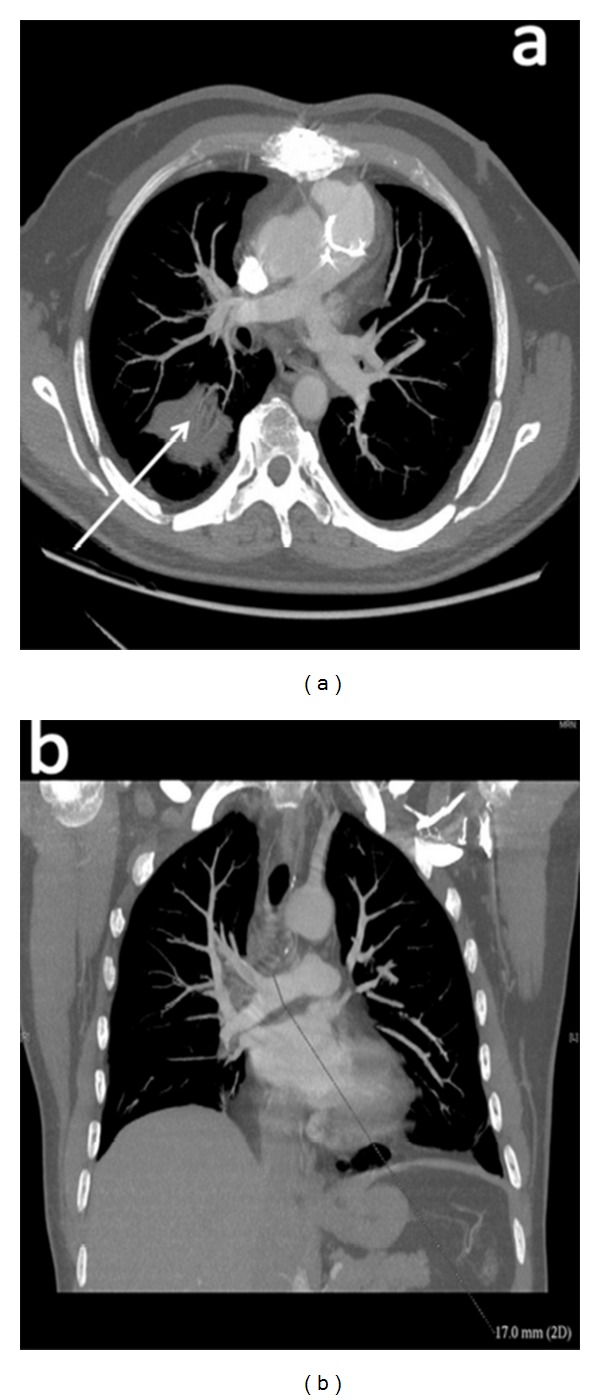
Postoperative axial (a) and coronal (b) CTPA showing the patent right pulmonary artery tree and residual lung consolidation (arrow) 5 days after surgery.

The patient improved substantially after surgery with reduction of all inflammatory markers and fading of the sepsis. He was put on a six-week IV antibiotics course and eventually went home after completion of the antibiotics course. The sixteen-week follow-up CTPA showed widely patent right pulmonary tree (Figure 2).

## 4. Discussion

Isolated pulmonary valve endocarditis is a rare condition present only in 2% of all hospitalized patients with infective endocarditis [1]. Main risk factors are intravenous drug abuse, alcoholism, sepsis, and infected venous pacing system [2]. The diagnosis of pulmonary valve endocarditis is clinically challenging and tricky. Clinical manifestations commonly affect the respiratory system owing to pulmonary septic embolization. High-grade maintained fever and marked increase of serum inflammatory markers differentiate septic pulmonary embolism due to the right-side infective endocarditis from nonseptic pulmonary embolism [3]. It is difficult to diagnose without clinical suspicion. This delays appropriate treatment; thus careful medical history and physical examination by the physician are very important. Also, thorough echocardiographic evaluation of all cardiac valves, including right-sided valves, should be carried out in all patients with suspecting infective endocarditis, especially when the patient has a risk factor [4]. Due to the low sensitivity of TTE for evaluating the pulmonary valve (vegetations are only identified by TTE in 70% of the cases), TOE is the next step in the diagnostic workup after blood cultures being positive [2]. 

There is an established evidence-based guideline for left-sided heart valve endocarditis [5], but this is not the case with regards to the right-side heart valve endocarditis due to the rarity. This, in turn, makes the timing of surgical intervention unclear. Previous case reports concluded that the proper timing of surgery in right-side infective endocarditis should be determined based on the type of organism (especially staphylococcus aureus and gram-negative bacilli), secondary heart failure, severe valvular dysfunction, presence and extent of local invasion, pulmonary involvement such as septic emboli and cavitation, and systemic involvement such as disseminated intravascular coagulopathy or acute renal failure [6]. However, no definite guidelines are present to support the decision making, the team has to rely on their experience and patient's clinical condition in the decision making [7].

In conclusion, the learning points from this case include the following: (a) Follow-up for patients with history of congenital heart disease is required even if the malformation spontaneously healed, (b) high level of suspicion should be adopted in patients with unexplained pulmonary symptoms, especially those with positive blood cultures, (c) careful TOE examination of all heart valves is mandatory if endocarditis is suspected to visualize valve lesions or vegetation if any, and (d) prompt treatment of right-sided endocarditis will prevent complications such as massive septic pulmonary embolization.
